# Migrant Workers in Kazakhstan: Gender Differences in HIV Knowledge and Sexual Risk Behaviors

**DOI:** 10.1007/s10461-014-0914-9

**Published:** 2014-10-08

**Authors:** Baurzhan Zhussupov, Louise-Anne McNutt, Louisa Gilbert, Assel Terlikbayeva, Nabila El-Bassel

**Affiliations:** 1 Global Health Research Center of Central Asia, Columbia University, New York, NY USA; 2 Department of Epidemiology and Biostatistics, University at Albany School of Public Health, Rensselaer, NY USA; 3 102, Luganskogo str., Almaty, 050059 Kazakhstan

**Keywords:** HIV, Kazakhstan, Gender, Migrants, Mobility, Propensity score

## Abstract

This study compares sexual risk behaviors among male and female migrant market vendors in Almaty, Kazakhstan. From the Barakholka Market, 209 male and 213 female market vendors were randomly recruited. Self-reported data were collected through standardized face-to-face interviews. Dry blood spot was used as specimen for syphilis testing. Propensity score stratification was used to estimate adjusted prevalence or rate ratios by gender. Compared to male migrant workers, females had lower HIV knowledge and were less likely to have multiple sexual partners. There was no evidence of a gender difference for prevalence of syphilis, condom use with unsteady partners, and safe sex communication between couples. Associations between mobility patterns and engagement in multiple sexual partnerships were stronger among women than men. Efforts should be made to mitigate the gender differential in HIV knowledge among migrants, especially women. Such efforts need to be implemented in both home and host countries.

## Introduction

Kazakhstan is one of only seven countries that experienced a 25 % or more increase in HIV incidence from 2001 to 2009 [1]. Injecting drug use is the main driver of the HIV epidemic in Eastern Europe and the Central Asian region [2]. In addition to injecting drug users, the HIV epidemic in Kazakhstan is concentrated among the sexual partners of injecting drug users and sex workers, who then spread the virus to other population groups including youth and migrants [3, 4]. About 7 % of the HIV cases registered in Kazakhstan in 2009 were migrants from abroad [5]. In Tajikistan, more than 9 % of registered HIV cases were among labor migrants returning from other countries [6]. The populous and highly mobile nature of migrants in the region suggests that they contribute to the spread of infection while being difficult to reach with educational prevention messages.

Ranked 15th in the world list of top migrant-receiving countries, Kazakhstan is second only to Russia for hosting migrants from other Central Asian countries [7]. In Central Asia, Kyrgyzstan, Tajikistan, and Uzbekistan represent possible source countries with estimated numbers of international migrants and percentages of the population they constitute as follows: 223,000 (4.1 %); 284,000 (3.8 %) and 1,176,000 (4.2 %), respectively [7]. Internal migration in Kazakhstan is also significant. The official data reveals that 15 % of the country population changed a residence within the country in a period of 10 years [8]. The risk of HIV infection among both domestic [9] and foreign [10] labor migrants is elevated due to various social, economic, and political factors: many migrants are separated from their families; have limited access to HIV prevention information and services because of language and legal barriers, and often have much lower levels of education compared to citizens of their host countries [11, 12]. Migrants from Central Asia also tend to have low HIV knowledge [13, 14]. Although substance use is not prevalent among migrants, a quarter of male migrants engage in risky sexual behavior [13].

Trade-related internal and external movements are a special type of migration. Trading is one of the main activities of migrants in addition to construction, agriculture, and services. Both men and women are involved in migration for commercial migration work in contrast with construction work, which is predominately male [15]. While there are no accurate statistics about gender distribution among Kyrgyz, Tajik, and Uzbek on labor migrants living and working in Kazakhstan some estimates show that Tajik and Uzbek female migrants range between 10 and 40 %. Nearly 50 % of Kyrgyz migrant workers are women [15].

As in the general population, male migrants are more likely to engage in risky sexual behavior when compared to female migrants [16]. Sexual double standards, which exist worldwide, cause different views on acceptable sexual behaviors according to gender. Women are expected to abstain from sexual contact outside of marriage. In contrast, promiscuous behavior among men is generally tolerated or even expected [17]. Woman’s risk of HIV remains elevated despite her commitment to a monogamous relationship if her male partner engages in risky sexual behaviors. Finally, female migrants may be more vulnerable to HIV infection due to the risks associated with sexual exploitation and abuse [18].

In a previous publication from this study, we found that migrant vendors in Kazakhstan showed positive associations between mobility characteristics and sexual risk behaviors [19]. In the current paper, we examine the impact of gender differences on sexual risk behaviors, HIV knowledge, and safe sex communication between couples. Such knowledge will inform targeted health risk education strategies among this difficult-to-reach population in an effort to curb the increasing incidence of HIV in Central Asia.

We hypothesize that gender effect on the following dependent variables including multiple sexual partners, condom use, syphilis sero-status, HIV knowledge, and safer sex communication skills. To analyze this hypothesis we use the propensity score model to assess the effect of gender on our dependent variables. While there have been no studies investigating the effect of gender on these variables in Kazakhstan before, previous research carried out elsewhere inform our hypotheses:

- In South Africa, in comparison with male migrants, women migrants are likely to have fewer sexual partners and report lower levels of condom use [20].
- In West Africa, 10–20 % fewer women possess comprehensive HIV knowledge in comparison with men [21].
- In the United States, the ratio of male to female syphilis cases is 1.8 to 1 [22].
- Finally, safer sex communication among sexual partner is associated with condom use [23] and is an important component of women having control of their sexual lives [24].

The paper also examines the role of gender as effect modifier for four key independent variables—the number of times traveled in the past year to buy goods, the number of times traveled the past year to visit friends or family, the number of years employed at the market, and job status (stall owner or vendor). We want to know if the effects of these variables on the likelihood of having multiple partners are the same for men and women. The mobility and migration variables were guided by previous research.

## Methods

### Sample

From July 2007 to October 2007, total of 422 market vendors—209 males and 213 females—from the Barakholka Market in Almaty, Kazakhstan were randomly selected for participation in the study. Data were collected through standardized in-person interviews. Internal or external migrants aged 18 years and older were eligible for participation, if they reported engaging in vaginal or anal sex in the past 90 days, maintained a residence (home) outside of Almaty or outside of Kazakhstan, and were employed as a worker or owner in a randomly selected stall at the Barakholka Market.

### Measurement

#### Socio-Demographics

Data was gathered for the following socio-demographic variables: gender, age, education level, country of citizenship, type of residency, and living together with spouse or girlfriend/boyfriend.

#### HIV Knowledge

Participant knowledge about HIV was measured through a series of 12 yes or no questions about HIV prevention, transmission, and treatment, which were coded into a one-item scale with values varying from 0 to 12. The HIV knowledge score was calculated as the number of correct responses to these questions.

#### Safe Sex Communication

We assess safe sex communication with a seven-item summary scale, based on the respondents’ answers (“yes” coded as 1) to the following questions:

In the past 90 days have you and your steady partner(s) discussed:

1. The need to use condoms?
2. The need to get tested for HIV, Hepatitis C and/or other sexually transmitted disease?
3. Oral sex as an alternative to vaginal or anal sex?
4. Masturbation as an alternative to vaginal or anal sex?
5. How to prevent HIV?
6. How to prevent sexually transmitted disease?
7. How to make safer sex more fun?

The scale is a sum of responses with possible answers ranging from 0 to 7, with higher scores corresponding to increased levels of communication about safe sex. The score had a Cronbach’s alpha of 0.83, indicating good internal validity.

#### Mobility

Mobility was characterized by the number of times traveled in the past year to buy goods to sell at the Barakholka Market, the number of times traveled to visit friends or family in the past year.

#### Work-Related Characteristics

The number of years employed at the market, and primary job responsibility at market stall (owner or vendor) were used to measure work-related characteristics.

#### Biotest for Syphilis

A capillary whole blood, blotted on to a filter paper, was used as the specimen for testing for syphilis and HIV. Syphilis is known to be prevalent in Central Asia and serves as an important indicator of risky sexual behavior, past or present. We did find one instance of HIV infection but chose to omit it from the analysis and discussion since it is not possible to carry out any statistical testing with a single positive case. Serologic testing for syphilis was performed by the Republican AIDS Center’s reference laboratory in Almaty using a standard immune-capture enzyme immunoassay (ICE Syphilis, Abbott Murex Diagnostics).

### Statistical Analyses

Prevalence ratios were computed to investigate the gender effect on binary outcome variables (having multiple partners, consistent condom use with an unsteady partner, and a positive syphilis test), and rate ratios were employed to identify associations between gender and the two outcome variables of interest, HIV knowledge and safer sex communication. Prevalence ratios were also used to address the second question about the effect of gender on associations between the mobility patterns of migrant workers and risky sexual behaviors. Statistical analyses were conducted in R version 2.14.2.

Stratification by the propensity score was used to adjust the propensity score. Propensity score analysis (PSA) was utilized to control for imbalances between gender groups, i.e., to mitigate gender-selection bias. A more precise multivariate method than logistic regression, PSA is widely used for covariate adjustment [25]. The propensity model included the variables that were considered to be possibly associated with gender or with outcomes. Nine socio-demographic and migration variables were included in the logistic regression model to calculate propensity scores as the predicted probability of being in the female group. The 20, 40, 60, and 80 % quintiles of the propensity scores were defined. Based on these quintiles, participants were stratified into five equal-size groups. Five groups were selected to achieve substantial gender group overlap in all groups, and the creation of five or more groups has been shown to reduce at least 90 % of the selection bias in observed covariates used to calculate propensity score [26].

The proportion of missing responses in covariates didn’t exceed 1.5 %; to estimate the missing data values, we used multiple imputations (MICE package, R). For the evaluation of balance diagnostic, we used Cohen’s h effect size for two proportions. The stratum-specific means were used to compute the model-adjusted value of the outcome variable in female and male groups. Bootstrap simulation was used to estimate the standard error for each parameter.

To assess the potential for missed confounders, a sensitivity analysis was performed to describe how the gender effect on outcome variables changes in response to an unobserved covariate with gender and outcome [27]. We defined the values of such associations that eliminated the gender effect on outcomes completely.

### Ethics Statement

The institutional review boards of Columbia University and the Ethics Review Board of the Ministry of Health of Kazakhstan approved protocols of the study.

## Results

### Balance Diagnostics

The 20th, 40th, 60th and 80th percentiles of the propensity score were 0.338, 0.435, 0.517, and 0.715, respectively. The proportion of females within each stratum ranged from 12.2 to 80.2 %. Thus, our model fit the crucial requirement of propensity score overlap between female and male respondents in all groups. The baseline characteristics of female and male market vendors are described in Table 1. Five of the nine variables had standardized differences in excess of 0.20, indicating the presence of systematic differences in baseline characteristics between female and male respondents. The standardized differences ranged from 0.00 to 0.59 with a mean of 0.18. After propensity score adjustment, standardized differences decreased to a mean of 0.07 with a range of 0.01–0.19. The balance diagnostics demonstrated that female and male participants reached similar distributions of the baseline covariates.

**Table 1:** Baseline characteristics of the study sample

	N = 422	%	Unadjusted data			Propensity score model		
			Females (N = 213) % (*n*)	Males (N = 209) % (*n*)	Effect size*	Females	Males	Effect size*
Age group								
< 21 years	40	9.5	5.6 (12)	13.4 (28)	0.27	8.6	9.2	0.02
21–25	95	22.5	16.9 (36)	28.2 (59)	0.27	23.9	22.0	0.05
26–30	145	34.4	39.9 (85)	28.7 (60)	0.24	38.5	34.3	0.09
31+	142	33.6	37.6 (80)	29.7 (62)	0.17	29.0	34.5	0.12
Education								
Secondary or less	53	12.9	8.6 (18)	17.4 (35)	0.27	15.9	13.3	0.07
High school	244	59.5	61.7 (129)	57.2 (115)	0.09	54.3	55.9	0.03
More than high school	113	27.6	29.7 (62)	25.4 (51)	0.10	27.9	26.9	0.01
Country of citizenship								
Kazakhstan	153	36.3	38.2 (81)	34.4 (72)	0.08	40.0	36.9	0.06
Kyrgyzstan	120	28.5	30.7 (65)	26.3 (55)	0.10	28.7	31.0	0.05
Uzbekistan	97	23.0	27.8 (59)	18.2 (38)	0.23	22.0	19.6	0.06
Other	51	12.1	3.3 (7)	21.1 (44)	0.59	8.8	12.5	0.12
Type of residence								
Own apartment	26	6.2	5.2 (11)	7.2 (15)	0.08	7.9	7.5	0.02
Family home/apartment	32	7.7	6.2 (13)	9.2 (19)	0.11	6.5	8.9	0.09
Partner’s home/apartment	12	2.9	2.9 (6)	2.9 (6)	0.00	5.0	4.2	0.04
Someone else’s home/apartment	316	75.8	72.9 (153)	78.7 (163)	0.14	72.0	75.4	0.08
Other	31	7.4	12.9 (27)	1.9 (4)	0.46	7.6	3.4	0.19
Spouse or girlfriend/boyfriend lives with participant								
Yes	238	56.7	61.6 (130)	51.7 (108)	0.20	53.0	57.9	0.10
No	182	43.3	38.4 (81)	48.3 (101)	–	47.0	42.1	–
Owner is primary responsibility at stall								
Yes	37	8.8	6.6 (14)	11.1 (23)	0.16	10.3	9.9	0.01
No	383	91.2	93.4 (199)	88.9 (184)	–	89.7	89.4	–
Travel in past year to purchase goods to sell at market								
Yes	153	36.4	39.2 (83)	33.7 (70)	0.11	42.0	40.3	0.03
No	267	63.6	60.8 (129)	66.3 (138)	–	58.0	59.7	–
Travel out of Almaty to visit family/friends in past year								
3+ times	200	48.1	55.0 (116)	41.0 (84)	0.28	54.4	47.9	0.13
<3 times	216	51.9	45.0 (95)	59.0 (121)	–	45.6	52.1	
Length of time in job at market								
≤1 year	33	7.8	6.6 (14)	9.1 (19)	0.09	5.8	7.1	0.05
>1 & ≤3 years	183	43.5	45.5 (97)	41.3 (86)	0.08	42.2	45.1	0.06
>3 years	205	48.7	47.9 (102)	49.5(103)	0.03	52.1	47.8	0.09

* Standardized difference of proportions

### Estimated Gender Effects

Our hypothesis is that five outcome variables characterizing HIV risk factors are affected by gender: (1) HIV and condom use knowledge, (2) syphilis, (3) multiple sexual partners in the past 90 days, (4) consistent condom use with an unsteady partner or partners in the past 90 days, and (5) safer sex communication among couples. The effects of gender on outcome variables, unadjusted and adjusted by PSA, are shown in Table 2.

**Table 2:** Gender effect on outcome variables (crude and propensity score model)

	Unadjusted data			Propensity score model		
	Female (N = 213)	Male (N = 209)	Prevalence/rate ratio (95 % CI)	Female	Male	Prevalence/rate ratio (95 % CI)
More than one sexual partner (%)	20.3	66.0	0.31 (0.23–0.41)	19.6	62.5	0.31 (0.20–0.45)
Consistent condom use with unsteady partner in past 90 days (%)	23.8	29.3	0.81 (0.44–1.49)	18.9	25.0	0.77 (0.29–1.41)
Biotest for Syphilis (%)	6.6	4.8	1.37 (0.62–3.02)	4.9	4.7	1.03 (0.28–2.65)
HIV and condom use knowledge (score mean)	2.5	3.3	0.75 (0.63–0.91)	2.7	3.5	0.77 (0.61–0.95)
Couple safer sex communication (score mean)	0.8	1.5	0.59 (0.42–0.84)	1.0	1.3	0.77 (0.51–1.10)

Women were less likely than men to have two or more partners in the past 90 days: 20.3 % of women compared to 66.0 % of men (prevalence ratio [PR] = 0.31, 95 % confidence interval [CI] = 0.23–0.41). In the adjusted propensity score model, the gender difference remained statistically significant with the same magnitude (PR = 0.31, CI = 0.20–0.45). There was no significant difference between males and females in their use of a condom consistently in the past 90 days in either the unadjusted rate (PR = 0.81, CI = 0.44–1.49) or the propensity score model (PR = 0.77, CI = 0.29–1.41). One reason might be the relatively small number of participants reporting unsteady partners (43 females and 138 males).

No statistically significant difference was detected for gender with respect to the prevalence of syphilis: 6.6 % of females and 4.4 % of males tested positive for syphilis (PR = 1.37, CI = 0.62–3.02). The difference remained insignificant after PSA (PR = 1.03, CI = 0.28–2.65). Female migrants demonstrated lower HIV and condom use knowledge, compared with male migrants both in the sample (rate ratio [RR] = 0.75, CI = 0.63–0.91) and the propensity score model (RR = 0.77, CI = 0.61–0.95). Similarly, the mean score for safer sex communication between couples was lower among female respondents than among male respondents (RR = 0.59, CI = 0.42–0.84), but statistical significance was diminished after propensity score adjustment (RR = 0.77, CI = 0.51–1.10).

### Sensitivity Analysis

A sensitivity analysis was performed for two statistically significant gender effects on having multiple sexual partners as well as HIV and condom use knowledge. A gender effect on having multiple sexual partners would be eliminated if an unobserved covariate with 50 % prevalence among males had an exceptionally strong association with gender (odds ratio [OR] = 55) and with having multiple partners (OR = 74). To eliminate the gender effect on HIV and condom use knowledge, an unobserved covariate with 50 % prevalence among males would need to have a strong association with gender (OR = 4.5) and with HIV and condom use knowledge (RR = 2.2). These results demonstrate that a presence of an unknown factor significantly related to both gender and the number of sexual partners is highly unlikely. However, our results show that the presence of such a factor connecting with both gender and HIV knowledge is more plausible.

### Gender as an Effect Modifier

Gender modifies mobility patterns as well as job characteristics with respect to sexual risk behaviors, particularly having multiple sexual partners (Table 3). Two mobility patterns—traveling in the past year to purchase goods to sell at the market and travelling out of Almaty to visit family and friends in the past year—were significantly associated with having multiple sexual partners among women but not among men.

**Table 3:** Association between having more than one sexual partner in the past 90 days with migration and job characteristics, stratified by gender

	Female			Male		
	>1 partner % (*n*)	1 partner % (*n*)	Prevalence ratio (PR) (95 % CI)	>1 partner % (*n*)	1 partner % (*n*)	Prevalence ratio (PR) (95 % CI)
Owner is primary responsibility at stall						
Yes	28.6 (4)	71.4 (10)	1.45 (0.60–3.49)	65.2 (15)	34.8 (8)	0.98 (0.72–1.35)
No	19.7 (39)	80.3 (159)	1.0	66.3 (122)	33.7 (62)	1.0
Travel in the past year to purchase goods to sell at the market						
Yes	32.5 (27)	67.5 (56)	2.60 (1.50–4.53)	67.1 (47)	32.9 (23)	1.02 (0.83–1.25)
No	12.5 (16)	87.5 (112)	1.0	65.9 (91)	34.1 (47)	1.0
Travel out of Almaty to visit family/friends in the past year						
3+	30.2 (35)	69.8 (81)	3.55 (1.73–7.27)	67.9 (57)	32.1 (27)	1.04 (0.85–1.26)
<3	8.5 (8)	91.5 (86)	1.0	65.3 (79)	34.7 (42)	1.0
Number of years working at market						
≤1 year	7.1 (1)	92.9 (13)	1.0	57.9 (11)	42.1 (8)	1.0
1–3 years	19.6 (19)	80.4 (78)	2.74 (0.40–18.92)	72.1 (62)	27.9 (24)	1.25 (0.83–1.87)
>3 years	22.8 (23)	77.2 (78)	3.19 (0.47–21.80)	63.1 (65)	36.9 (38)	1.09 (0.72–1.64)

## Discussion

Migrant market vendors have a higher prevalence of reporting multiple partners compared to the general population. The likelihood of having multiple partners was threefold higher among male migrants when compared to female migrants. In 2007, sexual intercourse with more than one partner within the past 12 months was reported by 25 % of Kazakh men and 5 % of Kazakh women aged 15–49 (UNGASS indicator 16) [28].

While having multiple sex partners contributes to the HIV epidemic when the HIV prevalence among the general population is high [29], this study showed the high potential for HIV transmission among migrant males and females, establishing them as a risk group of importance to epidemic control. Presence of sexual risk in both sexes was confirmed by high prevalence rates of syphilis among both male and female migrants.

Although the prevalence of multiple sexual partnerships was less common among women when compared to men, when additional sexual partnerships did occur among women they tended to be in conjunction with mobility and length of time worked at the market. This phenomenon should be considered in the design and accounted for in the development and implementation of HIV prevention strategies for migrant women.

Female migrant vendors had less HIV knowledge than male migrant vendors. In some countries, men typically demonstrate a better understanding. For instance, of the 85 countries providing data to UNAIDS about the proportion of young adults age 15–24 years who answered HIV knowledge questions correctly (UNGASS indicator #13), 22 countries reported better HIV knowledge among males than females (differential exceeding 5 %), and 12 countries reported the reverse [27]. The gender difference in HIV knowledge found in this study may be explained by factors originating in the home countries before migration. In countries, from which the migrants come, discussing sexuality is taboo, particularly for girls and women. For example, migrants’ wives in Tajikistan said that sexual behaviors, HIV/AIDS knowledge, condom use, and HIV testing were not subjects of communication with the husband [30].

Estimated gender effects are subject to several limitations. First, the study was observational and as such, residual, unidentified confounders may be unaccounted for in the analyses. However, the sensitivity analysis showed that existence of a covariate that could eliminate the gender effect on having multiple partners and HIV knowledge is unlikely. Second, the data are cross-sectional and we cannot identify the time when syphilis infection occurred. Thus, the values of potential confounders used in our analysis could differ from ones at the time syphilis was contracted. Third, the study was conducted in one city, Almaty, which reduces the generalizability of our results. Finally, we may expect some measurement bias to persist due to the influence of social desirability on reporting. Participants could avoid socially undesirable answers, e.g., reporting drug use or having multiple partners. However, the interviewers were trained and the questionnaire was tested to reduce the latter bias.

The findings from this research have important implications for research and prevention efforts among migrant workers in Central Asia, and in particular, for female migrants. Despite more than 30 years into the HIV epidemic, HIV research among migrant workers in Central Asia remains limited. We are not aware to any large scale epidemiological HIV research or prevention research targeting migrant female workers in the Central Asia. Although male migrant workers are typically the main focus for prevention efforts due to their greater likelihood of risky sexual and drug use behaviors and higher levels of mobility, female migrants are rarely included in research and prevention services. There is need for HIV epidemiological and prevention research among this population. Given that mobility plays a significant role, prevention strategies need to integrate these issues. The findings from this research underscore the need to increase HIV prevention for women in both their home and host countries. Moreover, empowering women to access services for HIV should be a key component of strategic public health efforts.
